# Tracheal Agenesis: A Challenging Prenatal Diagnosis—Contribution of Fetal MRI

**DOI:** 10.1155/2015/456028

**Published:** 2015-03-02

**Authors:** Charline Bertholdt, Estelle Perdriolle-Galet, Pascale Bach-Segura, Olivier Morel

**Affiliations:** ^1^Pôle de Gynécologie-Obstétrique, CHU de Nancy, Université de Lorraine, 10 rue du Docteur Heydenreich, 54 000 Nancy, France; ^2^Unité INSERM U 947, Nancy, Université de Lorraine, CHU de Brabois, Tour Drouet, rue du Morvan, 54511 Vandoeuvre-lès-Nancy, France; ^3^Pôle d'Imagerie Médicale, CHU de Nancy, Université de Lorraine, 10 rue du Docteur Heydenreich, 54 000 Nancy, France

## Abstract

Tracheal agenesis is a rare congenital anomaly. The prevalence is less than 1 : 50 000 with a male to female ratio of 2 : 1. This anomaly may be isolated but, in 93% of cases, it is part of polymalformative syndrome. The most evocative diagnosis situation is the ultrasonographic congenital high airway obstruction syndrome. Dilated airways, enlarged lungs with flattened diaphragm, fetal ascites and severe nonimmune hydrops can be observed. In the absence of a congenital high airway obstruction syndrome, the antenatal diagnosis of tracheal agenesis is difficult. Tracheal agenesis should be suspected in the presence of an unexplained polyhydramnios associated with congenital malformations. The fetal airway exploration should then be systematically performed by fetal thoracic magnetic resonance imaging. A case of Floyd's type II tracheal agenesis, detected during the postnatal period, is reported here. The retrospective reexamination of fetal magnetic resonance images showed that the antenatal diagnosis would have been easy if a systematical examination of upper airways had been performed. Prenatal diagnosis of tracheal agenesis is possible with fetal MRI but the really challenge is to think about this pathology.

## 1. Introduction

Tracheal agenesis is a rare congenital anomaly characterized by complete or almost complete failure of trachea development. The prevalence is less than 1 : 50 000 with a male to female ratio of 2 : 1. A case of Floyd's type II of tracheal agenesis, detected during the postnatal period, is reported here.

## 2. Case Report

A 21-year-old women, gravida 1, para 0, was referred at 17 weeks' gestation because of the existence of a complex fetal heart anomaly. A double-outlet right ventricle was associated with a malposition of the great arteries, a ventricular septal defect, and a pulmonary artery stenosis.

A single umbilical artery and costovertebral abnormalities were also detected at ultrasound.

Fetal CT scan (computerized tomography scan) revealed abnormal vertebral angulation, hemivertebra, and rib anomalies.

The fetal medicine team, including a cardiopediatrician, discussed both diagnosis and prognosis, and the couple clearly expressed their wish to continue the pregnancy.

At fetal MRI (magnetic resonance imaging), performed at 31 weeks of gestation, no craniocerebral anomaly and no evocatory signs of CHARGE syndrome [1] (mnemonic term for coloboma, heart defects, choanal atresia, retarded growth and development, genital abnormalities, and ear anomalies) were found. Amniocentesis provided a normal XY karyotype. Prenatal ultrasound at 32 weeks showed polyhydramnios and suggested esophageal atresia but it was not confirmed. At 35 weeks, the fetus was eutrophic and the polyhydramnios was stable. Because of fetal heart abnormalities involving any immediate postnatal cardiovascular pediatric care, a C-section was performed at 39 weeks.

The newborn, a male infant weighting 2780 G, presented a severe respiratory distress immediately after birth. Ventilation was impossible and attempts of tracheal intubation were unsuccessful. The larynx visualization was correct, but the tube did not advance beyond the vocal cords and a high resistance was felt under the glottis. Emergency tracheotomy was performed and a complete tracheal agenesis was found at surgical exploration. A complete interruption of the trachea beyond the vocal cords associated with a tracheoesophageal fistula at the level of the carina was found at fibroscopic examination.

The ventilation of the newborn was temporarily possible through the tracheoesophageal fistula via an esophageal intubation. The newborn unfortunately died within 5 hours after birth.

The retrospective analysis of fetal MRI images showed complete absence of the trachea.

**Figure 1:**
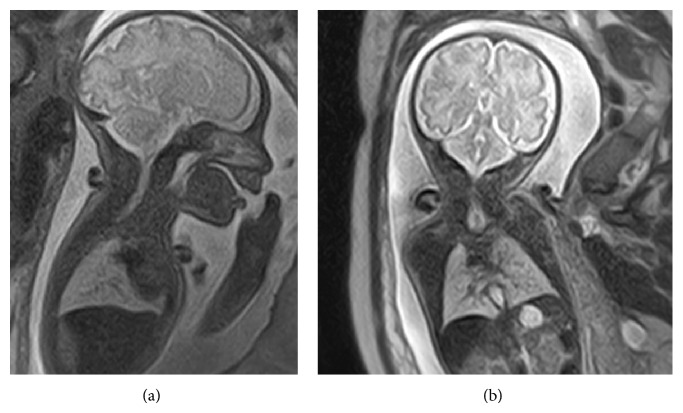
Case report: fetal MRI. Saggittal (a) and axial (b) view. Absence of continuity between subglottic area and bronchial bifurcation: Floyd's classification of type II.

The upper airways, normally visible as high intensity signal in T2-weighted image [2], were stopped below the glottis and only bronchial bifurcation was present (Figure 1).

The diagnosis of a Floyd's type II [3] tracheal agenesis was confirmed at pathological examination.

## 3. Discussion

Tracheal agenesis is complete or almost complete failure of trachea development. Payne first described this condition in 1900 and less than 200 cases have been published worldwide [4]. In 1962, Floyd et al. [3] classified tracheal agenesis in three anatomic types with incidence of 13%, 65%, and 22%, respectively [5]. Type I is characterized by agenesis of the proximal trachea. In type II, there is complete agenesis of trachea and the carina communicates with the oesophagus before it bifurcates. Type III represents complete agenesis of the trachea and the bronchi originate individually of the distal part of oesophagus.

Tracheal agenesis can be suspected in the presence of a CHAOS (congenital high airway obstruction syndrome) [6]. The syndrome presents with bilaterally enlarged echogenic lungs, which are due to intrapulmonary liquid retention caused by tracheal agenesis. Flattened diaphragm and ascites can be observed. The presence of associated tracheoesophageal fistula, however, allowed here the evacuation of intrapulmonary liquid and therefore the CHAOS was not present.

In our case, the antenatal diagnosis could have been realized even in the absence of CHAOS but* a priori* with fetal MRI only. The upper airway, indeed, was visualized till bronchial bifurcation at MRI, presenting with a high intensity signal in T2 because of its liquid content [2]. The diagnosis could then be proposed in the presence of a lack of continuity. In our images, the trachea was not visible, whereas glottis and bronchial bifurcation were clearly present.

In the majority of the cases, a polyhydramnios is observed. The initial bring up diagnosis is usually oesophageal agenesis which displays the same ultrasound appearance.

In 93% of the cases of tracheal agenesis, associated congenital malformations that may correspond to polymalformative syndrome such as VACTERL syndrome (vertebral anomalies, anal atresia, cardiovascular anomalies, tracheoesophageal fistula, renal anomalies, and limb defects), Fraser syndrome (tracheal agenesis, radial anomalies, complex heart malformations, and duodenal atresia), or the TARCD syndrome (cryptophthalmos, syndactyly, laryngeal atresia, and urogenital malformation) are observed [7].

In our case, the combination of polyhydramnios and congenital anomaly was present. Because of the presence of a tracheoesophageal fistula, however, the diagnosis of tracheal agenesis was not evoked. This lethal malformation could have been demonstrated prenatally as shown in retrospective reading of MRI images. MRI interpretation was performed by the study radiologist highly specialized in the field of fetal imaging. The antenatal diagnosis was missed because the eventuality of a tracheal agenesis was not raised.

To our knowledge, this is the second reported case of antenatal MRI images of fetal tracheal agenesis without CHAOS [8].

Prenatal diagnosis of tracheal agenesis is feasible with fetal MRI provided that a highly trained fetal radiologist performs the examination. The real challenge, however, remains to think about it in the absence of CHAOS.
